# Quit outcomes among clients ineligible for cessation medication through the state quitline: a retrospective, observational study

**DOI:** 10.1186/s12889-018-5923-6

**Published:** 2018-08-10

**Authors:** Adrienne B. Lent, Patrick A. O’Connor, Ryan C. Reikowsky, Uma S. Nair, Melanie L. Bell

**Affiliations:** 1 Health Promotion Sciences, University of Arizona, Mel and Enid Zuckerman College of Public Health, 1295 N Martin Ave, Tucson, AZ 85724 USA; 2 Biostatistics, University of Arizona, Mel and Enid Zuckerman College of Public Health, 1295 N Martin Ave, Tucson, AZ 85724 USA; 3 Epidemiology and Biostatistics, University of Arizona, Mel and Enid Zuckerman College of Public Health, 1295 N Martin Ave, Tucson, AZ 85724 USA

**Keywords:** Smoking, Tobacco, Quitline, Medicaid, Telephone counseling, Cessation medication

## Abstract

**Background:**

Distribution of tobacco cessation medications through state quitlines increases service utilization and quit outcomes. However, some state quitlines have moved to models in which callers are instructed to obtain quit medications through their health insurance pharmaceutical benefit. We aimed to investigate the impact of this policy on medication access and quit outcomes in the state quitline setting for clients who must obtain covered medications through the state Medicaid program. We hypothesized that clients with Medicaid who were referred by their healthcare provider would be more likely to report using quit medication and have higher quit rates compared to clients with Medicaid who engaged the quitline on their own.

**Methods:**

An observational, retrospective study was conducted using state quitline clients with Medicaid health insurance who were ineligible for quitline provided cessation medications. Clients were stratified by referral type: self-referred, passively referred, and proactively referred. Unadjusted and adjusted logistic regression was used to estimate the effect of referral type on both quit status and cessation medication use.

**Results:**

Proactively referred clients were less likely to use quit medication (53.6%) compared to self (56.9%) and passively referred clients (61.1%). Proactively referred clients had lower quit rates (31.4%), as compared to passively referred (36.0%) and self-referred (35.1%). In adjusted models, proactively referred clients were significantly less likely to be quit than passively referred clients (OR = 0.75, 95% CI: 0.56, 0.99). There were no statistically significant differences in medication use or number of coaching sessions among proactive, passive, and self-referred clients in adjusted models.

**Conclusions:**

In adjusted models, medication use did not significantly differ by mode of entry in this population of Medicaid beneficiaries. Psychosocial factors such as intention to quit in the next 30 days, social support for quitting, education level, race, and ethnicity impacted quit status and differed by mode of entry. Quitlines should use tailored strategies to increase engagement and reduce barriers among proactively referred clients.

**Electronic supplementary material:**

The online version of this article (10.1186/s12889-018-5923-6) contains supplementary material, which is available to authorized users.

## Background

Smoking is the leading preventable cause of premature death and disease in the United States [1]. Smoking rates have decreased significantly since the release of the first Surgeon General’s Report on Smoking and Health in 1964 but remain disproportionately high among low income individuals [2, 3]. Tobacco use rates for Medicaid beneficiaries are nearly double the privately insured population and 11% of Medicaid expenditures ($22 billion) nationally are attributable to smoking-related diseases [4, 5].

Tobacco cessation medications, including over-the-counter nicotine replacement therapy (NRT), are effective in helping people quit. Using NRT, even without behavioral counseling, can increase the likelihood of cessation by 50–70% [6]. In the state quitline setting, distribution of nicotine replacement therapy at no cost to clients results in higher client quit rates when compared to clients who do not receive quitline-provided medication [7, 8]. Therefore, access to cessation medications plays an important part in quitting smoking.

Quitlines are an effective, accessible, and evidence-based public health approach to delivering quit medication support as well as behavioral counseling [9, 10]. Access to NRT, while facilitated in the quitline setting, has been somewhat impeded with reductions in state quitline funding. Some state quitlines (such as North Carolina and Alabama) have begun to restrict services to individuals who can receive NRT through their insurance plan, while continuing to provide services to high-risk populations (e.g., uninsured) who do not have access to covered quit medication treatments outside the state quitline setting [11]. Most health insurers are required to cover tobacco cessation treatment as a preventive service under the Affordable Care Act, which includes all seven Food and Drug Administration (FDA) approved quit medications without copays or prior authorization [12]. Therefore, some state quitlines have moved to models in which callers are instructed to obtain quit medications through their health insurance pharmaceutical benefit. While navigating callers to an external health system for quit medications may decrease quitline costs, the impact of this policy on quit medication access and quit outcomes, especially among the high-risk Medicaid population, in the state quitline setting is unknown. While previous studies have examined quit rates and medication utilization among clients when NRT is distributed through the state quitline [7, 8, 13, 14], little is known about quit medication access and the impact on cessation rates among quitline clients who are instructed to obtain health plan covered quit medications external to the state quitline.

Further, while most health insurers cover quit medications, they are often underutilized. The structure of obtaining insurance-covered cessation medications may pose a barrier to access since members must get a prescription from their healthcare provider and fill it at an in-network pharmacy. An in-network pharmacy is a pharmacy that is contracted with a health plan to provide pharmacy services to that health plan’s members. While obtaining a prescription may be easy for those with private insurance, it may be more difficult for low-income individuals with Medicaid who may experience delays in care in an often understaffed healthcare system [15, 16]. Additionally, a lack of awareness of health insurance cessation medication benefits may contribute to low utilization of covered quit medications; however, evidence suggests that advice from a healthcare provider to quit is an important predictor of cessation medication use, especially among Medicaid members [17].

Medicaid beneficiaries in Arizona who enroll in state quitline services are ineligible for quitline provided NRT. Instead, these clients are instructed to obtain a prescription through a healthcare provider to secure quit medications covered by their health plan. Using a sample of Medicaid quitline clients in Arizona, this paper examined whether cessation medication use and quit rates vary between clients who were referred or advised to enroll in services by their healthcare provider compared to those self-initiating contact with the quitline. Data for this study were obtained from Medicaid beneficiaries enrolled in the Arizona Smokers’ Helpline (ASHLine). This study was limited to Medicaid beneficiaries in Arizona since data were obtained from Arizona’s state quitline. Clients ineligible for quitline provided NRT who want medication are required to obtain insurance covered quit medications with a prescription that must be filled at a pharmacy. Therefore, we hypothesized that clients with a direct connection to a healthcare provider would be more likely to use quit medications and subsequently have higher quit rates than those who engaged the quitline on their own. We hypothesized that the mechanism for increased access to cessation medication among clients is connection to a healthcare provider. Furthermore, there is research that shows that medication use and quit rates differ by all three quitline modes of entry [18]. Our primary objective was to examine differences in cessation medication use and our secondary objective was to examine differences in quit rates. Our exploratory objective was to test whether cessation medication use mediated the effect of referral type on quit status, if there was evidence that referral type was associated with cessation medication use and quit rates.

## Methods

### Study design, setting and participants

The Arizona Smokers’ Helpline (ASHLine), Arizona’s state-funded tobacco quitline, provides free phone-based behavioral counseling to all Arizona residents. ASHLine provides no-cost NRT to clients not covered by Arizona’s state Medicaid program (i.e., uninsured and privately insured). ASHLine does not provide free NRT to those who are covered by Arizona’s Medicaid insurance since this state program covers twelve weeks of the seven FDA-approved cessation medications twice a year. ASHLine educates and assists clients with Medicaid with navigating the process of obtaining insurance-covered medications, which includes getting a prescription from their primary care provider and filling it at an in-network pharmacy.

**Fig. 1:**
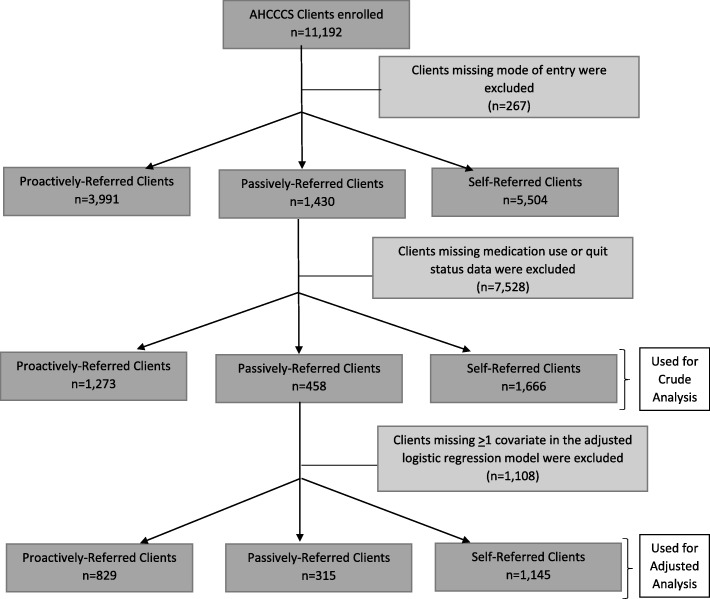
Flow Diagram

A retrospective analysis was performed using ASHLine data for clients who reported having Medicaid health insurance and who were enrolled in tobacco cessation services between January 2011 and June 2016. Descriptive data were collected during the client intake survey at the time of enrollment into the program. A trained, external evaluation team collected self-reported medication use and quit status through a telephone survey at seven months after program enrollment as a part of standard quitline evaluation practices recommended by the North American Quitline Consortium (NAQC) [19]. During the study period, 49,284 individuals enrolled in ASHLine and 11,192 (22.8%) had Medicaid. Those who were missing mode of entry (*n* = 267) and medication use or quit status (*n* = 7528) data were excluded. The final sample size used for crude analyses was 3397 = 30% of the original sample; for adjusted analysis the sample size was 2289 clients (20% of the original sample). Figure 1 shows the criteria used to select the clients included in the crude and adjusted analyses.

### Measures

Our primary outcomes of interest were (a) medication use, which was defined as self-reporting using any of the seven FDA-approved cessation medications (nicotine gum, nicotine patch, nicotine lozenge, nicotine inhaler, nicotine nasal spray, Varenicline (Chantix), and Bupropion (Zyban)) during a client’s quit attempt, and (b) quit status at seven months post program enrollment, defined as self-reporting not having used tobacco within the last 30 days. The independent variable was referral type (proactive, passive, and self). Proactively referred clients were contacted by the quitline after receipt of an electronic or fax referral from their healthcare provider. Passively referred clients self-initiated contact with the quitline on their own after receiving advice from a healthcare provider. Self-referred clients also self-initated a call to the quitline but did so after seeing an advertisement or hearing about the program from a friend or family member. Self and passive referral status were identified at enrollment by asking clients how they heard about ASHLine. Controlling variables gathered at program enrollment included clients’ self-reported: age; gender (female, male); education (high school or less, some college or more); race (White, Black, Asian, American Indian, multi-racial, other); diagnosis of a chronic condition including asthma, cancer, chronic obstructive pulmonary disorder, diabetes, heart disease, and hypertension (yes, no); mental health condition including depression, bipolar, schizophrenia, anxiety, and alcohol or drug abuse (yes, no); intensity of physical addiction to nicotine as measured by the commonly used valid and reliable Fagerstrom Test for Nicotine Dependence (measured on a 0–10 scale with higher scores indicating greater dependence) [20–23]; social support to quit smoking measured by a five-point Likert scale; presence of other smokers in the home (yes, no); and confidence to quit smoking for at least 24 h (dichotomized as not confident or somewhat confident versus confident, very confident, or extremely confident). Number of completed coaching sessions was assessed after program completion.

#### Statistical methods

ANOVA and chi-square tests were used to evaluate differences in baseline and seven month follow-up factors stratified by referral type (proactive, passive, and self). Unadjusted and adjusted logistic regression was used to estimate the effect of referral type on both quit status and cessation medication use at seven month follow-up. Adjusted models included the variables listed above, were pre-specified; based on background knowledge; literature review that show they are associated with smoking behavior; and our own extensive experience with these data [18, 24–27]. These variables are collected by ASHLine as a part of their intake process and are based on the minimal data set for state quitlines as outlined by NAQC [28]. Odds ratios (OR) and 95% confidence intervals (CI) were estimated. The fit of each model was evaluated using the Hosmer-Lemeshow test [29]. The assumption of linearity in the logit was assessed for continuous variables using restricted cubic splines [30]. Baseline characteristics of clients with seven month follow-up were compared with those without follow-up.

#### Exploratory analysis

We also planned to test whether cessation medication use mediated the effect of referral type on quit status, if it was found that referral type was associated with cessation medication and quit rates. Mediation analysis aims to quantify the relative direct and relative indirect effects of an exposure (referral type) on an outcome (quit status) through an intermediary variable (cessation medication use), known as the mediator. The *relative direct effect* is the effect the exposure has on the outcome while controlling for the mediator. All analyses were performed using SAS version 9.4 (SAS Institute, Cary, North Carolina).

## Results

### Client characteristics by mode of entry

Compared to self and passively referred clients, proactively referred clients were more likely to be male, non-white (Black, Asian, American Indian, multi-racial, other), Hispanic, have a high school education or less, and report having other smokers in the home. Compared to proactively referred clients, self and passively referred clients were more likely to smoke more cigarettes per day, have higher nicotine dependence levels, have more social support, and intend to quit in the next thirty days. Passively referred smokers were more likely to have a chronic condition compared to self and proactively referred clients (Table 1). Proactively referred clients were less likely to use quit medication (53.6%) compared to self (56.9%) and passively referred clients (61.1%). Proactively referred clients had lower quit rates (31.4%), as compared to passively referred (36.0%) and self-referred (35.1%).

**Table 1:** Descriptive statistics (*N* = 3397). Mean (SD) shown for continuous variables, frequencies (%) shown for categorical variables

Characteristics	Referral type			*P*-value
	Self (*n* = 1666)	Passive (*n* = 458)	Proactive (*n* = 1273)	
Age (years)	48.7 (13.0)	50.1 (11.6)	49.1 (12.9)	0.11
Gender				**0.02**
Male	572 (34.4)	158 (34.7)	494 (39.1)	
Female	1089 (65.6)	298 (65.4)	768 (60.9)	
Race				0.01*
White	1211 (81.6)	327 (79.6)	782 (76.9)	
Black or AA	144 (9.7)	44 (10.7)	138 (13.6)	
Asian	6 (0.4)	3 (0.7)	11 (1.1)	
American Indian	40 (2.7)	7 (1.7)	38 (3.7)	
Multiracial	45 (3.0)	17 (4.1)	18 (1.8)	
Other	38 (2.6)	13 (3.2)	30 (3.0)	
Education				**< 0.0001**
High school or less	834 (50.1)	241 (52.6)	782 (61.4)	
Some college or more	832 (49.9)	217 (47.4)	491 (38.6)	
Hispanic	251 (18.2)	66 (17.3)	309 (31.8)	**< 0.0001**
Children living in the household	435 (31.1)	107 (28.1)	334 (33.2)	0.17
Family size	2.4 (1.7)	2.5 (1.8)	2.6 (1.8)	**0.03**
Chronic condition	1066 (66.7)	327 (74.5)	886 (72.1)	**0.01**
Mental health condition	951 (59.9)	273 (63.5)	725 (59.5)	0.33
*Baseline tobacco use behaviors*				
Other smokers in the home	626 (46.8)	176 (47.6)	523 (52.5)	**0.02**
Smoking allowed in the home				0.05
Not allowed	731 (52.3)	202 (52.2)	482 (47.7)	
Allowed in some places	221 (15.8)	67 (17.3)	201 (20.2)	
Allowed anywhere	446 (31.9)	118 (30.5)	325 (32.2)	
Number of cigarettes smoked per day, mean (SD)	16.6 (10.1)	17.6 (11.7)	14.7 (8.9)	**< 0.0001**
Nicotine dependence, mean (SD)	5.0 (2.3)	5.1 (2.3)	4.6 (2.2)	**0.01**
*Other baseline factors*				
Number of quit attempts during past 12 months, mean (SD)	2.8 (7.8)	2.8 (7.5)	2.0 (3.4)	**0.01**
Confidence to quit (for at least 24 h)				0.56
Poor, fair	237 (16.8)	68 (17.4)	186 (18.5)	
good, very good, excellent	1170 (83.2)	323 (82.6)	818 (81.5)	
Intention to quit (in the next 30 days)				**< 0.0001**
No, I don’t know	41 (3.6)	11 (2.8)	80 (7.8)	
Yes, I have already quit	1369 (96.4)	388 (97.2)	945 (92.2)	
Social support				**0.01**
Poor, fair	357 (25.3)	75 (19.3)	272 (27.4)	
good, very good, excellent	1055 (74.7)	313 (80.7)	720 (72.6)	
*Post-baseline factors*				
Reach rate**	58.0%	56.4%	58.5%	0.42
Number of coaching sessions before 7-month follow-up				0.92
0–4	994 (59.7)	278 (60.7)	764 (60.0)	
5+	672 (40.3)	180 (39.3)	509 (40.0)	
Days in program	85.1 (83.5)	80.7 (76.8)	79.1 (67.5)	0.11
Others smokers at home (7-month follow-up)	506 (30.8)	156 (34.5)	418 (33.4)	0.19
Medication use	948 (56.9)	280 (61.1)	682 (53.6)	**0.01**
Quit	585 (35.1)	165 (36.0)	400 (31.4)	0.06

Boldface indicates statistical significance (*p* < 0.05)

^*^ Fisher’s Exact Test used for comparisons

^**^ Reach rate is calculated using data for all clients with a mode of entry (*n* = 10,925)

### Adjusted odds of quit status and medication use

Unadjusted and adjusted results were similar (Table 2), although odds ratios comparing referral types were changed slightly, with marginally significant comparisons becoming non-significant and vice versa. We report the adjusted results here. Proactively referred clients were significantly less likely to be quit than passively referred clients (OR = 0.75, 95% CI: 0.56, 0.99). There was no evidence of difference in quit status between self-referred and passively referred clients (OR = 1.12, 95% CI: 0.85, 1.47) or between self-referred and proactively referred clients (OR = 0.84, 95% CI: 0.68, 1.02). Variables that negatively affected quit status included self-reported mental health condition (OR = 0.70, 95% CI: 0.58, 0.85) and nicotine dependence score (OR = 0.92, 95% CI: 0.88, 0.95). Number of coaching sessions (OR = 2.87, 95% CI: 2.38, 3.45) and a client’s intention to quit tobacco in the next 30 days (OR = 2.29, 95% CI: 1.41, 3.71) significantly increased the odds of being quit.

**Table 2:** Odds ratios, 95% confidence intervals, and *p*-values for the outcomes of quit status and medication use

	Quit Status		Medication use	
Unadjusted Model	OR (95% CI)	*p*-value	OR (95% CI)	*p*-value
Referral type				
Self-referral	Ref		Ref	
Passive referral	1.04 (0.84, 1.29)	0.72	1.19 (0.96, 1.47)	0.10
Proactive referral	0.85 (0.73, 0.99)	**0.04**	0.87 (0.76, 1.01)	0.07
Proactive vs Passive	0.81 (0.65, 1.02)	0.07	0.73 (0.59, 0.91)	**0.01**
Adjusted Model				
Referral type				
Self-referral	Ref		Ref	
Passive referral	1.12 (0.85, 1.47)	0.42	1.10 (0.85, 1.43)	0.46
Proactive referral	0.84 (0.68, 1.02)	0.08	0.91 (0.75, 1.09)	0.29
Proactive vs Passive	0.75 (0.56, 0.99)	**0.05**	0.82 (0.63, 1.08)	0.15
Age (per 10 years)	0.93 (0.86, 1.01)	0.07	1.08 (1.01, 1.16)	**0.02**
Gender				
Female	Ref		Ref	
Male	1.05 (0.87, 1.28)	0.59	0.90 (0.75, 1.07)	0.23
Education				
High school or less	Ref		Ref	
Some college or more	1.14 (0.95, 1.37)	0.17	1.37 (1.16, 1.63)	**0.01**
Chronic condition				
No	Ref		Ref	
Yes	1.00 (0.81, 1.22)	0.96	1.21 (1.00, 1.46)	0.05
Mental health condition				
No	Ref		Ref	
Yes	0.70 (0.58, 0.85)	**0.01**	0.96 (0.81, 1.15)	0.69
Fagerstrom test for nicotine dependence	0.92 (0.88, 0.95)	**<.0001**	1.04 (1.00, 1.08)	**0.04**
Number of coaching sessions before 7 month follow-up				
0–4	Ref		Ref	
5+	2.87 (2.38, 3.45)	**<.0001**	1.32 (1.11, 1.57)	**0.01**
Social support				
Good, very good, excellent	Ref		Ref	
Poor, fair	0.98 (0.80, 1.21)	0.87	1.10 (0.91, 1.34)	0.32
Other smokers in the home				
No	Ref		Ref	
Yes	0.98 (0.81, 1.17)	0.79	0.96 (0.81, 1.14)	0.62
Intention to quit (in the next 30 days)	Ref		Ref	
No, I don’t know	2.29 (1.41, 3.71)	**0.01**	1.37 (0.94, 1.98)	0.10
Yes, I have already quit				

Values in bold are statistically significant at the 0.05 level

In the adjusted model (unlike the unadjusted model), medication use did not differ significantly among the three groups, with OR = 1.1 for passively versus self-referred clients (95% CI: 0.85, 1.43); OR = 0.91 for proactively versus self-referred clients (95% CI: 0.75, 1.09); and OR = 0.82 (95% CI, 0.63, 1.08) for proactive versus passively referred clients. Higher education (OR = 1.37, 95% CI: 1.16, 1.63), a higher nicotine dependence score (OR = 1.04, 95% CI: 1.00, 1.08), and receiving 5 or more coaching sessions (OR = 1.32, 95% CI: 1.11, 1.57) were significantly associated with a client using medication. A 10-year increase in age increased the odds of using medication during the quit attempt (OR = 1.08, 95% CI: 1.01, 1.16).

### Missing data and dropout

Baseline comparisons of clients included in the adjusted analysis with clients with missing data (either follow-up or one of the covariates from the adjusted models) are shown in Additional file 1: Table S1. We report statistically significant comparisons, but advise that the large sample size can make very small differences statistically significant. Compared to clients in the final adjusted models, those who dropped out or had incomplete data were slightly younger, slightly more likely to be male, slightly more likely to have children living in their household, slightly more likely to have a chronic condition, slightly more likely to have other smokers in the home, slightly more likely to have a higher frequency of tobacco use, have slightly more quit attempts in the past 12 months, and have slightly less social support.

### Mediation

Mediation results are not given, as the evidence for association between referral type and medication and cessation rates was not strong.

## Discussion

### Key findings

The purpose of this study was to examine differences in medication utilization and quit outcomes by mode of entry in a high-risk, low-income group of clients who are ineligible for quitline provided NRT (i.e., ASHLine clients with Medicaid insurance). The hypothesis that ASHLine Medicaid clients who were referred by a healthcare provider (proactive and passive) would have higher medication use rates and subsequently higher quit rates compared to clients who were self-referred was not strongly supported. Although there were significant differences in medication use across proactive, passive, and self-referred clients in unadjusted models, upon adjusting, these differences were no longer significant. However, medication use overall in this sample was low (57%). This lack of variation in medication use across the three groups may be explained by overall low utilization of medication in this population, perhaps due to the increased barriers in obtaining a prescription from a health care provider (vs. the quitline providing/mailing out the medication). Proactively referred clients were less likely to quit compared to passively referred clients in adjusted models. The marginal unadjusted odds ratio in quit status between passively and self-referred clients decreased from 0.85 to 0.84, but became statistically non-significant in adjusted models. The client characteristics of proactively referred clients differed from those who engaged the quitline on their own and may play a more critical role in cessation compared to medication use. Proactively referred clients had a lower intention to quit in the next 30 days, social support for quitting, education level, and were more likely to be non-white, Hispanic. Therefore, proactively referred clients with Medicaid represent a high-risk group of tobacco users with unique barriers to quitting.

### Interpretation of results

Our analysis showed that factors other than medication use impacted the odds of quitting. Proactively referred clients with Medicaid differed from their passive and self-referred peers, which may explain the differences in quit outcomes. Proactively referred clients were twice as likely to report having no intention to quit in the next 30 days compared to passive and self-referred clients who initiate their own engagement with the quitline. Although intention to quit may help explain the difference in quit outcomes among passive and proactive referrals, it does not explain why the quit rates between proactive and self-referred clients were not found to be different after adjustment. It may be possible that passively referred clients are unique - they receive advice from a health care provider to quit tobacco (a known factor in influencing smoking behavior change [17]) and they self-initiate a call to the quitline (an indication of a high motivation to quit). This synergistic effect of health care provider messaging combined with a high motivation to quit may explain the differences in quit outcomes between passive and proactive clients. Additionally, quit rates between proactive and self referrals may not have differed in adjusted models because each group is only getting one effect: advice from a healthcare provider or a high motivation to quit. Additional factors that may contribute to the observed differences in tobacco cessation status are the baseline barriers to quitting associated with clients who are proactively referred. Our analysis of client characteristics by mode of entry suggests that proactively referred clients are more likely to be Hispanic, non-white individuals with social and environmental barriers to quitting (e.g., lack of social support, living with other smokers, and low education levels), which could negatively impact their likelihood of success.

### Relation to other literature

The findings from this study are consistent with other research that has shown proactively referred clients may be fundamentally different than clients who initiate their own engagement with the state. A study conducted by the Massachusetts state quitline, which offered free NRT to all callers, found that provider referred clients were more likely to be less educated, non-white (Black or other), not as ready to quit and have lower quit rates compared to self-referred clients [31]. Another study conducted by the Ohio state quitline found similar results indicating that clients proactively referred by a healthcare provider were less motivated to quit and educated compared to self-referred clients [32]. A recent study also found that proactively referred clients had different characteristics than from those who engaged the quitline on their own and were more likely to be non-white (Black or other), less educated, have less social support, and have other smokers in the home [18]. Therefore, proactively referred clients with Medicaid are a high-risk group who faces additional barriers to quitting. These client characteristics may be an important determination of cessation regardless of quitline provided NRT.

Quitlines could benefit from tailoring services and creating policies that are better suited to meet the unique needs of the proactively referred population. Quitline staff should use tailored strategies, such as harm reduction, for engaging clients who may have low intention or motivation to quit. Alternatively, behavioral strategies aimed at increasing motivation to quit, such as motivational interviewing with goal setting, should be incorporated and enhanced. While our study found that quit medication did not differ by mode of entry, quitlines should implement policies that support cessation for high-risk groups who may experience barriers in access to care and difficulty navigating complex health systems. Medicaid beneficiaries greatly underuse cessation medications compared to those with private insurance [17]. Nationally, only 10% of Medicaid beneficiaries who smoke fill a prescription for a tobacco cessation medication annually [33]. Although policies that limit the distribution of NRT to certain client groups may decrease quitline costs, reliance on external coverage of cessation medications for high-risk Medicaid smokers may add to barriers when quitting and underuse of cessation treatments.

While medication use did not differ across the three groups in adjusted models, it is important to note that just over half of our sample of quitline clients with Medicaid reported medication use (Table 1). This appears to be much lower than the overall medication use rate among all ASHLine clients, which was reported at 73% in a recent study [18]. Future research should compare medication use among quitline clients with Medicaid and those without Medicaid who receive quitline provided NRT to assess overall differences in medication use and quit rates. This would help further understand the impact of quitlines limiting NRT and having to rely on external insurers for medication coverage, especially among high-risk groups such as Medicaid beneficiaries.

#### Strengths and limitations

This study was conducted in a state quitline setting, which included a large sample size and identified the real-world implications of limiting tobacco treatment services to a high-risk population. These translational findings can inform other state quitlines looking to leverage externally provided treatment to residents. Medication use and quit rates were collected at seven month follow-up survey. Although our survey completion rates were low (41.5%) and represent a potential source of response bias, these response rates are common among state quitlines. While NAQC has established a goal of 50% follow-up, most state quitlines fall well below this recommendation since loss to follow-up is common [19]. Secondly, we did not assess adherence and usage of NRT. While clients may self-report using NRT at the end of program, we did not assess whether clients used their full supply or if they adhered to usage guidelines at follow-up. While this paper aims to assess access to quit medications and associated quit outcomes among Arizona’s Medicaid quitline client population, further research may examine adherence to medication use. Medication use and quit status were self-reported are not verified biochemically. However, self-reported smoking status has been shown to be reliable and is common practice in tobacco research [34]. It is also important to note that our study only assessed for intention to quit in the next 30 days and did not assess for motivation to quit.

## Conclusions

This study highlights differences in Medicaid client characteristics by mode of entry and tobacco cessation medication use and quit rates. Higher intention to quit, social support, education levels, and race and ethnicity may play an important role in quit rates. State quitlines should tailor services to address these factors and social determinants of health within high-risk, low-income smokers such as Medicaid beneficiaries. State quitlines and funding agencies should consider the implications of limiting quitline services in high-risk populations and implement policies that reduce barriers and support smoker engagement.

## Additional file

Additional file 1: **Table S1.** Descriptive statistics between AHCCCS clients analyzed (*N* = 3397) and AHCCCS clients dropped due to missing data (*N* = 7795). Mean (SD) shown for continuous variables, frequencies (%) shown for categorical variables. (DOCX 22 kb)

## Abbreviations

AHCCCS: Arizona Health Care Cost Containment System
ASHLine: Arizona Smokers’ Helpline
NAQC: North American Quitline Consortium
NRT: Nicotine Replacement Therapy
