# Supplementary material for: Quit outcomes among clients ineligible for cessation medication through the state quitline: a retrospective, observational study
Source: BMC Public Health. 2018 Aug 10;18:1001. doi: 10.1186/s12889-018-5923-6 (PMC6086054; doi:10.1186/s12889-018-5923-6)
Supplement: Supplementary file 1 — Table S1. Descriptive statistics between AHCCCS clients analyzed (N = 3397) and AHCCCS clients dropped due to missing data (N = 7795). Mean (SD) shown for continuous variables, frequencies (%) shown for categorical variables. (DOCX 22 kb) [file 12889_2018_5923_MOESM1_ESM.docx]

**Appendix**

Table A1. Descriptive statistics between AHCCCS clients analyzed (N=3397) and AHCCCS clients dropped due to missing data (N=7795). Mean (SD) shown for continuous variables, frequencies (%) shown for categorical variables.

| Characteristics | Complete data  (N=3397) | Incomplete data  (N=7795) | P-value |
| --- | --- | --- | --- |
| Referral type |  |  | 0.17 |
| Self | 1666 (49.0) | 3838 (51.0) |  |
| Passive | 458 (13.5) | 972 (12.9) |  |
| Proactive | 1273 (37.5) | 2718 (36.1) |  |
| Age (years) | 49.0 (12.8) | 44.8 (13.4) | **<0.0001** |
| Gender  Male  Female | 1224 (36.2)  2155 (63.8) | 2620 (33.9)  5108 (66.1) | **0.02** |
| Race  White  Black or AA  Asian  American Indian  Multiracial  Other | 2320 (79.7)  326 (11.2)  20 (0.7)  85 (2.9)  80 (2.8)  81 (2.8) | 5213 (77.9)  802 (12.0)  34 (0.5)  207 (3.1)  235 (3.5)  191 (2.9) | 0.11 |
| Education  High school or less  Some college or more | 1857 (54.7)  1540 (45.3) | 4316 (55.4)  3479 (44.6) | 0.49 |
| Hispanic | 632 (22.9) | 1439 (22.8) | 0.92 |
| Children living in the household | 876 (31.5) | 2218 (37.6) | **<0.0001** |
| Family size | 2.5 (1.8) | 2.7 (2.1) | **<0.0001** |
| Chronic condition | 2279 (69.8) | 4806 (64.3) | **<0.0001** |
| Mental health condition | 1949 (60.2) | 4448 (60.1) | 0.91 |
| ***Baseline tobacco use behaviors*** |  |  |  |
| Other smokers in the home | 1325 (49.0) | 3091 (53.6) | **<0.0001** |
| Smoking allowed in the home  Not allowed  Allowed in some places  Allowed anywhere | 1415 (50.6)  492 (17.6)  889 (31.8) | 3011 (50.7)  1132 (19.1)  1798 (30.3) | 0.17 |
| Age began tobacco, mean (SD) | 17.3 (6.3) | 17.1 (6.1) | 0.11 |
| Frequency of current tobacco use  Not at all  Some days  Every day | 239 (8.1)  56 (1.9)  2667 (90.0) | 369 (5.9)  97 (1.6)  5775 (92.5) | **0.01** |
| Number of cigarettes smoked per day, mean (SD) | 16.1 (10.0) | 17.1 (13.7) | **0.01** |
| Nicotine dependence, mean (SD) | 4.8 (2.3) | 4.9 (2.3) | 0.69 |
| ***Other baseline factors*** |  |  |  |
| Number of quit attempts during past 12 months, mean (SD) | 2.5 (6.5) | 3.8 (23.6) | **0.01** |
| Confidence to quit (for at least 24 hrs)  Poor, fair  good, very good, excellent | 491 (17.5)  2311 (82.5) | 1110 (18.8)  4781 (81.2) | 0.14 |
| Intention to quit (in the next 30 days)  No, I don’t know  Yes, I have already quit | 142 (5.0)  2702 (95.0) | 299 (5.0)  5715 (95.0) | 0.97 |
| Social support  Poor, fair  good, very good, excellent | 704 (25.2)  2088 (74.8) | 1629 (27.7)  4250 (72.3) | **0.01** |
| ***Post-baseline factors*** |  |  |  |
| Number of coaching sessions before 7-month follow-up  0-4  5+ | 2010 (59.2)  1387 (40.8) | 6393 (82.0)  1402 (18.0) | **<0.0001** |
| Days in program, mean (SD) | 82.3 (77.0) | 52.0 (53.0) | **<0.0001** |
| Others smokers at home (7-month follow-up) | 1080 (32.3) | 149 (31.7) | 0.81 |
| Medication use | 1910 (56.2) | 63 (64.9) | 0.09 |
| Quit | 1150 (33.9) | 275 (25.8) | **<0.0001** |

Boldface indicates statistical significance (p<0.05)
